# Unlocking the Potency of Keyhole Limpet Hemocyanin: Structural Insights, Immunological Mechanisms, and Therapeutic Frontiers

**DOI:** 10.3390/ijms27177921

**Published:** 2026-09-05

**Authors:** Paula Cermakova, Ondrej Cehlar, Juraj Piestansky

**Affiliations:** 1 Department of Galenic Pharmacy, Faculty of Pharmacy, Comenius University, Odbojarov 10, 832 32 Bratislava, Slovakia; cermakova36@uniba.sk; 2 Institute of Neuroimmunology, Slovak Academy of Sciences, Dubravska cesta 9, 845 10 Bratislava, Slovakia; ondrej.cehlar@savba.sk; 3 Department of Pharmaceutical Chemistry, Faculty of Pharmacy, Comenius University, Odbojarov 10, 832 32 Bratislava, Slovakia

**Keywords:** carrier protein, glycosylation, immunogenicity, immunotherapy, keyhole limpet hemocyanin, vaccine development

## Abstract

Keyhole limpet hemocyanin (KLH) is a large copper-containing glycoprotein derived from the marine gastropod *Megathura crenulata*. Originally functioning as an oxygen transport molecule, KLH has gained considerable attention in biomedical research due to its exceptional immunogenic and immunostimulatory properties. Its complex quaternary structure, extensive glycosylation, and xenogeneic origin contribute to its ability to induce robust humoral and cellular immune responses in mammals without significant toxicity. These characteristics have established KLH as one of the most widely used carrier proteins in vaccine development and as a valuable model antigen for the investigation of adaptive immune responses. This review summarizes current knowledge on the biological origin, molecular structure, biosynthesis, and post-translational processing of KLH, with particular emphasis on its unique glycan architecture and its contribution to immunogenicity. Advances in glycomic and structural analyses have revealed an extraordinary diversity of N-linked glycans that distinguish KLH from mammalian glycoproteins and play a central role in immune recognition. The review further discusses methods for KLH isolation, purification, and characterization, as well as its application in experimental and clinical immunology as a standardized tool for assessing antigen-specific immune responses. In addition, the therapeutic and translational potential of KLH is examined across multiple biomedical fields. Particular attention is given to its use as a carrier protein in conjugate vaccines, its role in cancer immunotherapy, and its emerging applications in the development of vaccines and immunotherapeutic strategies targeting neurodegenerative diseases, atherosclerosis, and substance use disorders. Collectively, the available evidence highlights KLH as a unique marine-derived biomolecule that bridges glycobiology, immunology, and translational medicine, and continues to serve as an important platform for the development of next-generation immunotherapeutics and vaccine technologies.

## 1. Introduction

The giant keyhole limpet, *Megathura crenulata*, is a marine gastropod mollusk belonging to the family *Fissurellidae*. It inhabits rocky coastal habitats along the Pacific Ocean, with a distribution ranging from Point Conception in California, USA, to Isla Asunción in Baja California Sur, Mexico [1]. The species is of socioeconomic relevance as a nutritional resource for human consumption, since limpets provide high-quality protein as well as essential vitamins (notably A and D) and minerals such as phosphorus and iron [2]. In addition, it has significant biomedical importance in the pharmaceutical sector due to its production of the highly immunostimulatory protein, KLH. In general, hemocyanins in mollusks are involved in various immunological and physiological functions, including respiration [3], innate immunity [4], antimicrobial activity, hemolytic activity, or phenoloxidase activity [5]. Because of the complex quaternary structure, extensive glycosylation pattern, and high molecular complexity, KLH cannot currently be fully reproduced by synthetic or recombinant approaches, and the protein is obtained primarily through extraction from the hemolymph of living *Megathura crenulata* specimens [6].

The biomedical history of KLH can be traced to immunochemical studies of hemocyanin from *Megathura crenulata* in the 1960s. Weigle [7] characterized the immunochemical properties of hemocyanin in 1964 providing early evidence of its antigenic properties. KLH was subsequently introduced into human immunological research, including assessment of immunocompetence, and Curtis et al. [8] demonstrated in 1970 that KLH could elicit measurable primary humoral, cellular, and delayed-type hypersensitivity (DTH) responses in humans. Subsequent studies demonstrated that KLH could reliably induce both antibody production and delayed-type hypersensitivity reactions, which led to its widespread adoption as a “neoantigen”—an antigen to which humans have no pre-existing immunity. This property made KLH a valuable experimental tool for dissecting primary immune responses under controlled conditions [8,9].

Despite these advances, detailed understanding of the protein structure of KLH remained limited for many years. Early work identified two distinct subunit types, but it was only later that researchers established the existence of two separate oligomeric isoforms. The terminology “KLH1” and “KLH2,” now commonly used to distinguish these isoforms, was introduced in 1994 [9].

The remarkable progress of modern medicine has been accompanied by the rapid development of biopharmaceuticals, with protein- and peptide-based therapeutics representing one of the fastest-growing classes of medicines due to their high specificity, potency and favorable safety profiles compared to conventional small-molecule drugs [10]. Recent advances in protein engineering and site-selective bioconjugation have further expanded this concept beyond antibodies, enabling the development of diverse protein-based conjugates with tailored pharmacokinetic and immunological properties [11]. Among these, carrier proteins play an essential role in conjugate technologies by enhancing the biological activity or immunogenicity of covalently linked molecules. Within this context, KLH represents an attractive alternative biological scaffold [12]. Besides its well-established role as an exceptionally potent immunostimulatory protein, KLH possesses numerous surface-exposed lysine residues (170 in KLH1 and 150 in KLH2) that enable efficient chemical conjugation with haptens, peptides and small molecules while preserving its structural integrity and strong immunogenicity [13].

In recent years, KLH has already been extensively employed as a carrier protein in experimental and clinical conjugate vaccines, where covalent attachment of poorly immunogenic antigens markedly enhances antigen presentation and adaptive immune response [14,15,16,17]. Although current applications of KLH are primarily focused on immunization strategies [18,19,20], continuing advances in protein conjugation chemistry and biologic drug design may further expand its utility as a versatile protein scaffold for the development of next-generation biopharmaceuticals, including conjugates carrying therapeutic peptides or other bioactive molecules.

## 2. Origin, Biological Function and Structural Organization of KLH

**Figure 1:**
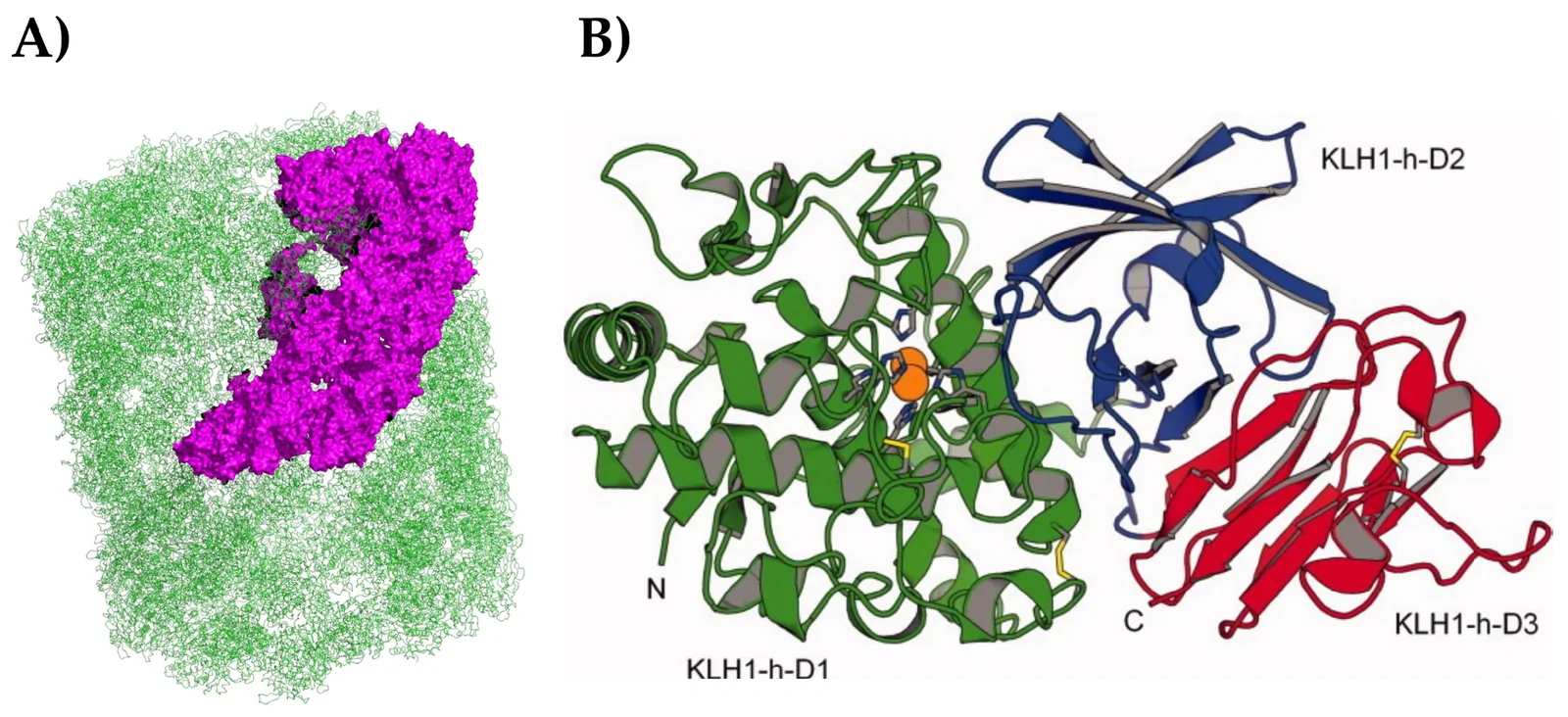
(**A**) Structure of KLH1 protein forming a didecamer. One dimer of KLH chains is highlighted as magenta surface. The remaining 18 chains are shown as green trace. The figure was created in Pymol 3.1 (Schrodinger LLC) using a structure with PBD ID 4BED. (**B**) KLH1-h subunit structure which is composed of three distinct domains, i.e., the N-terminal domain (KLH1-h-D1), the adjacent domain (KLH1-h-D2), and the C-terminal domain (KLH1-h-D3). The KLH1-h-D1 (green color) is typically composed of α-helices and harbors the cooper active site type 3. The KLH1-h-D2 (blue color) is represented by β-sandwich and covers the entrance to the active site. The KLH1-h-D3 (red color) has a cupredoxin-like fold, but without the active site. Reprinted with permission from: [19].

Hemocyanins are cylindrical, copper-containing molecules that act as oxygen transporting proteins for many mollusk species. This copper-dependent oxygen binding is responsible for the characteristic color change of hemocyanin: oxygenated forms appear blue due to Cu^2+^, whereas deoxygenated forms contain Cu^+^ and are colorless [21]. KLH is an extremely large molecule (~8000 kDa) comprising a variable number of subunits, KLH1 (390 kDa) and KLH2 (350 kDa), which share approximately 60% sequence identity at the protein level [6]. Both genes produce large, heavily glycosylated proteins of around 3400 amino acids. Each subunit is a large polypeptide composed of seven or eight globular regions known as functional domains or functional units (FUs) labeled *abcdefgh* [21]. Each FU is about 50 kDa in size, contains a binuclear copper active site responsible for oxygen binding and is glycosylated. These units differ considerably in their primary amino acid sequences. The modular arrangement of eight oxygen-binding FUs enables efficient oxygen transport while maintaining cooperative binding behavior. Simultaneously, the highly ordered didecameric assembly provides exceptional resistance to denaturation and proteolysis, properties that likely contribute to the long circulatory lifetime of hemocyanin in molluscan hemolymph. A particularly distinctive structural feature of molluscan hemocyanins is the presence of an internal collar complex located within the central cavity of the cylinder. In KLH1 this collar is formed primarily by FU-*g*, which assembles into five paired “arcs.” These arcs narrow the lumen of the cylinder and contribute substantially to overall structural rigidity. The additional FU-*h*, which is unique to gastropod hemocyanins, extends beyond the collar and forms a second structural element consisting of five paired “slabs” arranged as an annulus near one end of the cylinder. The first six functional units (FU-*a* to FU-*f*) were shown to constitute the cylindrical wall of the molecule. This architecture distinguishes gastropod hemocyanins from cephalopod hemocyanins, which lack FU-*h* entirely. The structure of KLH1 protein forming a didecamer is presented in Figure 1A. The landmark structural study by Gatsogiannis and Markl also comprehensively mapped 27 distinct intermolecular interface types, including wall–wall, collar–wall, slab–slab, slab–wall, and four decamer–decamer interfaces, explaining for the first time how the enormous quaternary assembly maintains both exceptional structural stability and the conformational flexibility required for cooperative oxygen binding. Their first near-complete 9 Å cryo-electron microscopy (cryo-EM) reconstruction and molecular model of the native KLH1 didecamer was presented in 2009 and resolved long-standing questions regarding the three-dimensional organization of this exceptionally large respiratory glycoprotein [22].

Harris et al. investigated the in vitro reassociation behavior of purified KLH1 and KLH2 subunits under controlled ionic conditions. Both subunits were shown to spontaneously reassemble into higher-order oligomeric and tubular structures, although with distinct morphologies: KLH1 formed flexible, open helical tubules, whereas KLH2 assembled into more stable, closed tubular forms. These findings highlight the structural plasticity of KLH and provide insight into the molecular basis of its heterogeneous supramolecular organization, which is relevant for its immunological and biotechnological applications [23]. Reassociation into higher-order structures is not restricted to the level of intact hemocyanin subunits. Proteolytically cleaved three- and four-FU fragments produced by treatment of KLH1 and KLH2 subunits with *S. aureus* V8 protease have the potential to reorganize and then reassociate exclusively into helical polymers rather than decameric oligomers.

In 2011, Jaenicke et al. published an atomic X-ray structure of KLH FU-h refined from the previously published model [24], however only with 4 Å resolution. This model brings atomic insights into the structure of KLH1 subunit that itself is composed of three domains (Figure 1B).

## 3. Biosynthesis and Post-Translation Processing of KLH

Biosynthesis of KLH occurs in specialized pore cells [9], which were further recognized as rhogocytes. They are distributed throughout the hemal and connective tissue, with the highest density of these cells occurring in the digestive gland. The study by Martin et al. demonstrated, that rhogocytes were able to synthesize KLH1 and also confirmed that the production of KLH is an ongoing process, and not a seasonal event [25]. In pulmonated gastropods, these cells are large metabolically active and characterized by an extensive rough endoplasmic reticulum (ER), reflecting their high capacity for protein synthesis. KLH is synthesized as a large polypeptide precursor on ribosomes bound to the rough ER. Following translation, the nascent protein enters the ER lumen, where it undergoes initial folding and N-linked glycosylation, a critical step for its stability and function.

Within the ER, KLH monomers begin to assemble into higher-order structures. Hemocyanin is known for forming very large cylindrical oligomers, and evidence from related gastropods suggests that these structures can form directly within ER cisternae. In some species, this assembly can even lead to the formation of paracrystalline arrays inside the cell. After initial assembly, KLH is transported through the Golgi apparatus, where further glycan processing and maturation occur. Once fully assembled and modified, the protein is secreted into the hemolymph, where it functions as an oxygen transport molecule. In contrast to other mollusks, the biosynthesis of KLH in *Megathura crenulata* appears to be less continuous. Early observations led to the suggestion that hemocyanin expression in *M. crenulata* may be seasonal [26]; however, subsequent evidence indicates that KLH1 biosynthesis is not restricted to a particular season. Martin et al. detected KLH-containing material in rhogocyte rough endoplasmic reticulum throughout the year, supporting an ongoing pattern of KLH1 production. Nevertheless, KLH1 mRNA was not detected in all rhogocytes at a given time, suggesting that KLH1 synthesis may occur asynchronously among individual rhogocytes rather than continuously and synchronously throughout the entire cell population [25]. Experimental observations suggest that production may occur in short, intermittent bursts, possibly influenced by environmental conditions or physiological stress. This intermittent synthesis may also explain the difficulty in detecting KLH or its mRNA in tissue samples. KLH biosynthesis involves coordinated processes of translation, glycosylation, folding, oligomerization, and secretion, with its extensive glycan content playing a central role in both structural integrity and biological activity [9].

Recent research also describes the possible stage at what the KLH begins to form in *M. crenulata*. The protein analysis mentioned in the paper by Thonig et al. [27] indicated presence of embryonic hemocyanin isoform in that keyhole limpet. However, the KLH material used for experimental, research and development, and clinical purposes is obtained from adult mollusks by a non-lethal method [28].

## 4. Glycan Structures and Immunogenic Epitopes of KLH

Glycans are structurally diverse carbohydrate moieties that are covalently attached to proteins and lipids or occur as free polysaccharides in biological systems. In glycoproteins, glycans significantly influence molecular stability, folding, solubility, intracellular trafficking, and biological recognition processes [29]. Their structural diversity arises from variations in monosaccharide composition, branching, linkage position, and stereochemistry, which collectively generate highly complex glycan architectures. Unlike proteins or nucleic acids, glycans are synthesized through non-template-driven enzymatic pathways, resulting in extensive microheterogeneity and dynamic biological functions. Glycans are therefore considered essential mediators of cell–cell communication, immune recognition, inflammation and host-pathogen interactions [30]. Alterations in glycosylation patterns are associated with numerous pathological conditions, including cancer, autoimmune diseases, and infectious disorders [31].

Within the context of KLH, glycans are of particular importance because they are directly linked to the exceptional immunogenic and immunostimulatory properties of this molecule. KLH glycans consist predominantly of N-linked oligosaccharides that differ substantially from mammalian glycosylation patterns. These glycans are believed to contribute significantly to the strong humoral and cellular immune responses induced by KLH, which explains its widespread use as a carrier protein, vaccine adjuvant and immunotherapeutic agent [32]. Glycosylation increases protein solubility and prevents their denaturation or aggregation. Thus, glycans have been described as an essential post-translational modification in terms of protein stability [33]. Although KLH is predominantly characterized by N-linked glycosylation, O-linked glycans have also been identified, specifically in the functional unit KLH2-c [5]. Detailed structural analyses of KLH glycans have revealed the presence of highly heterogenous population of neutral N-glycans. High-mannose N-glycans represent the predominant glycan population. *N*-Acetylglucosamine, *N*-acetylgalactosamine, galactose, mannose, and fucose were found in molar ratios of about 2.0:0.6:1.6:2.0:1.1. Another more uncommon glycan types include truncated oligomannose-derived glycans, glycans substituted with core fucose and core xylose and also glycans containing several structural motifs that have not been identified previously. Among the most characteristic structural features is the occurrence of Gal(β1-6)Man motifs, which are considered highly unusual in animal glycoproteins. Analysis of KLH also uncovered two additional previously unreported glycan modifications. The first involved the identification of a galactose residue attached directly to mannose through a β1,6 linkage on high-mannose and hybrid N-glycans. Some of these structures were further modified by the presence of an additional core α-fucose residue. The second novel feature was the discovery of a distinct glycan motif in which the α1,6-linked core fucose was substituted by a disaccharide extension consisting of two β1,4-linked galactose residues (Galβ1,4Galβ1,4Fucα1,6-). Although a single galactose residue linked to the inner core fucose had previously been reported in a molluscan protein, octopus rhodopsin, the occurrence of a core fucose substituted with two galactose residues represented a completely new structural finding [34].

KLH glycans contain fucosylated epitope structures that resemble glycan motifs found in parasitic organisms [35]. The study by Grzych et al. provides important insights into the immunological relationship between the parasite *Schistosoma mansoni* and KLH. It was found that KLH shares a cross-reactive carbohydrate epitope with 38 kDa antigen of *Schistosoma mansoni* the trematode parasite responsible for schistosomiasis. This antigenic similarity was demonstrated through multiple immunological approaches, including immunoprecipitation, Western blotting and inhibition assays, which collectively showed positive results [36]. This means that an immune response generated against KLH can also recognize and potentially protect a human from the parasite. Shared antigenic epitopes between different organisms can drive protective immune responses, highlighting the relevance of such mechanisms for vaccine development and immunotherapy, and supporting the use of KLH as a model antigen in immunological research.

Modern glycomic and glycoproteomic approaches, including mass spectrometry, high-performance liquid chromatography, and exoglycosidase sequencing, have substantially improved the characterization of KLH glycosylation. These analytical techniques enabled the identification of several novel glycan motifs and demonstrated that KLH possesses one of the most structurally complex glycosylation patterns described among molluscan glycoproteins. The unusual composition and high antigenicity of these glycans continue to make KLH an important model in glycobiology, immunology, and biopharmaceutical research [37].

## 5. Isolation, Extraction and Purification Strategies

The isolation procedure of KLH typically begins with the collection of hemolymph from *Megathura crenulata* specimens under cold conditions to minimize protein degradation and aggregation. The volume of hemolymph that can safely be extracted without adversely affecting the health of the source animal varies by species. Less than about 40% of the original animal weight by volume, calculated as the volume of hemolymph milliliters divided by the original animal weight in grams, has proven to be a reliable average for the gastropod *Megathura crenulata* [28]. Cellular debris and particulate contaminants are subsequently removed by low-speed centrifugation, yielding clarified hemolymph enriched in soluble hemocyanin. Early purification protocols relied primarily on preparative ultracentrifugation, exploiting the extremely high molecular mass of KLH oligomers, which sediment efficiently under high centrifugal forces. Ultracentrifugation remains one of the classical methods for obtaining native hemocyanin with high purity while preserving its oligomeric state [32].

**Figure 2:**
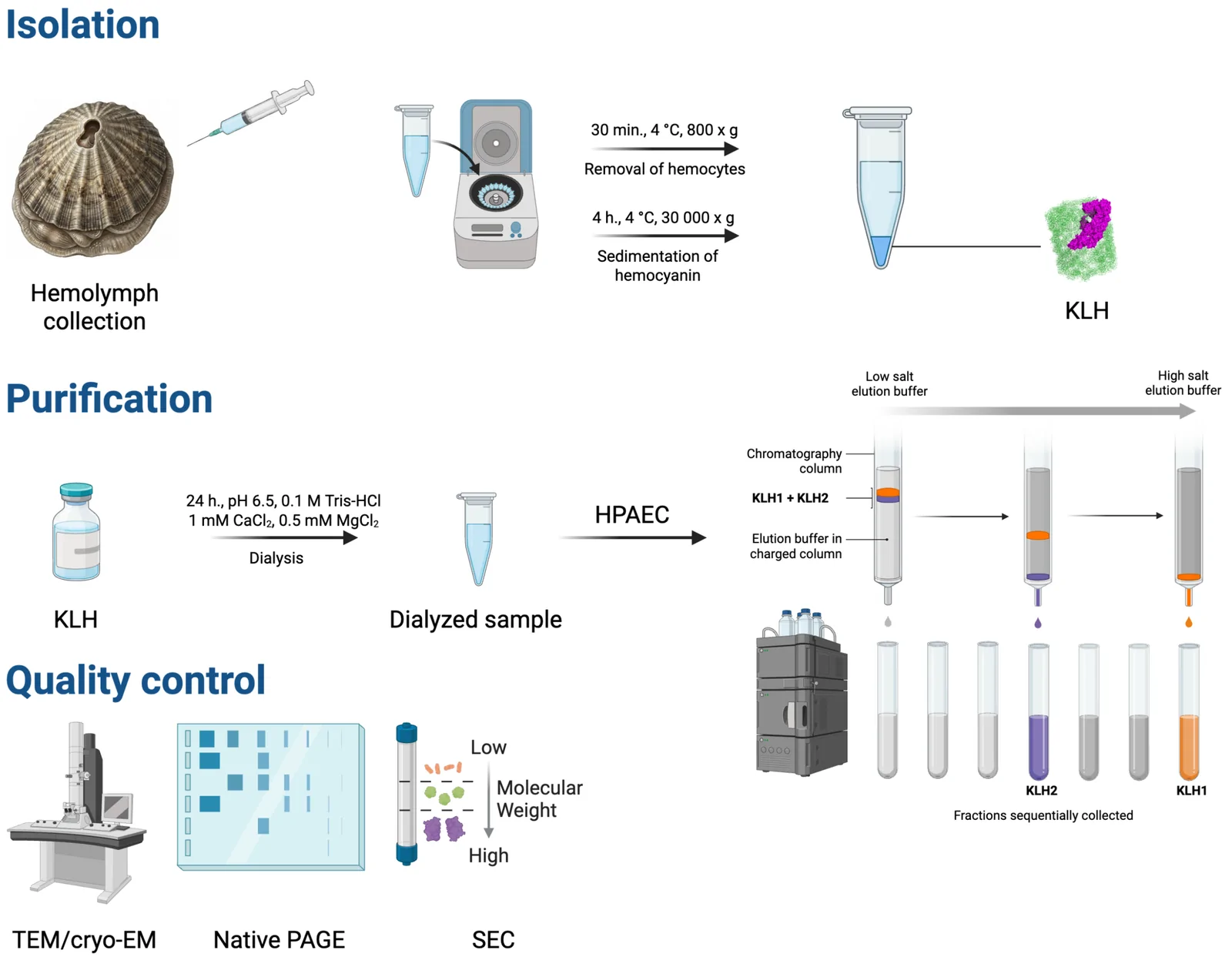
Isolation, purification and quality control of KLH. Created in BioRender. Cermakova, P. (2026) https://BioRender.com/v38j4hx, accessed on 4 September 2026.

Following the initial clarification step, further purification is commonly achieved using differential precipitation and chromatographic techniques. High-resolution purification of KLH is frequently performed using ion-exchange chromatography. Anion-exchange systems such as POROS HQ columns operate under carefully controlled pH and ionic strength conditions allow efficient separation of KLH1 and KLH2 subunits according to their charge differences [38]. Typically, from one keyhole limpet, it is possible to obtain pure KLH at the mean concentration level 5.45 g/L [39]. The whole process of isolation, purification and quality control of KLH can be seen in detail in Figure 2.

These chromatographic approaches significantly improve purity and facilitate structural and biochemical characterization of individual isoforms. Studies have demonstrated that KLH1 predominantly exists as stable didecamer, whereas KLH2 exhibits a greater tendency toward multidecamer formation and partial dissociation under mildly acidic conditions. Consequently, stabilization buffers containing divalent cations are routinely employed throughout purification to prevent structural disassembly.

Additional purification and characterization steps commonly include non-denaturing (native) gel electrophoresis of dissociated KLH1 and KLH2 subunits. Sodium dodecyl sulfate–polyacrylamide gel electrophoresis (SDS-PAGE) is of limited value because the two subunits have very similar molecular masses. Although preparative native PAGE has been employed to isolate the individual subunits, most purification strategies have focused on maintaining the integrity of the native oligomeric KLH complexes. Transmission electron microscopy (TEM) has played a major role in evaluating the preservation of native cylindrical didecameric structures during purification procedures and in monitoring the dissociation behavior of KLH2 under varying pH conditions [38].

Successful biochemical purification of intact KLH has relied heavily on TEM and crossed immuno-electrophoresis, applied in conjunction with chromatographic techniques such as ion exchange and gel filtration. Early observations also showed that animals maintained in saltwater aquaria for extended periods progressively lost KLH1, in some cases resulting in populations dominated by KLH2. These animals often exhibited signs of poor physiological condition, although the mechanism underlying selective KLH1 depletion remains unclear. One hypothesis is that KLH1 may be metabolized under conditions of negative protein balance to recycle amino acids and copper. Long-term captivity of *Megathura crenulata* (4–6 months up to over 1 year), as well as different husbandry systems (flow-through and recirculating seawater), did not significantly alter the KLH isoform profile, and no complete loss of either KLH1 or KLH2 was observed. Short-term food restriction produced only minor, variable individual effects without causing rapid or complete depletion of KLH1. In contrast, a supplemental diet was able to restore and increase KLH1 levels in animals that initially exhibited low amounts, returning isoform ratios to the natural range [40]. Notably, the KLH1:KLH2 ratio in pelleted hemocyanin varies substantially between individuals, with an average ratio of approximately 1:2 reported across 325 animals [6].

## 6. KLH as a Model Antigen for Evaluation of Adaptive Immune Responses

### 6.1. KLH as a T-Cell-Dependent Model Antigen

KLH is one of the most extensively used neoantigens for investigating adaptive immune responses in both preclinical immunotoxicology and clinical research. Because humans are not naturally exposed to KLH, immunization induces a well-defined primary antigen-specific immune response without interference from pre-existing immunological memory, making it an ideal model for studying T-cell-dependent (TD) immunity [41]. Consequently, KLH has become a valuable translational tool for evaluating immunocompetence, proof-of-mechanism, pharmacodynamic effects, and the biological activity of immunomodulatory therapies.

**Figure 3:**
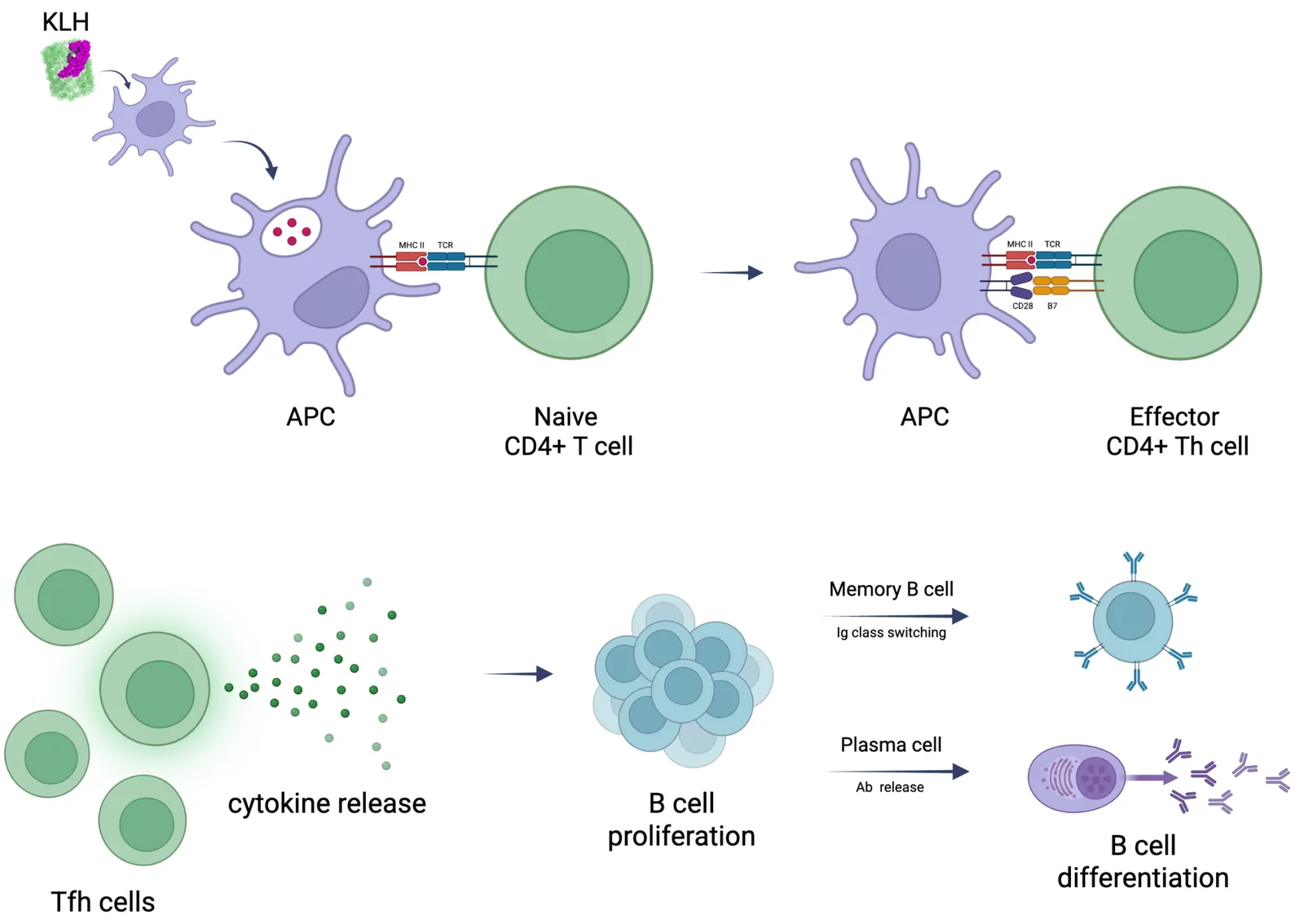
Mechanism of KLH-induced TDAR. Created in BioRender. Cermakova, P. (2026) https://BioRender.com/5kxxc1m, accessed on 4 September 2026.

The immune response elicited by KLH represents a classical T-cell-dependent antibody response (TDAR), which is considered the gold standard functional assay for assessing adaptive immune function in preclinical immunotoxicology. Following antigen uptake by antigen-presenting cells, KLH is processed and presented via major histocompatibility complex class II (MHC II) molecules to CD4+ T helper cells, resulting in coordinated activation of both cellular and humoral immunity [42]. This process encompasses antigen presentation, T-cell activation, B-cell proliferation, germinal center formation, immunoglobulin class switching, affinity maturation, and the generation of long-lived plasma cells and memory B cells (see Figure 3). Because the entire adaptive immune cascade is engaged, TDAR provides a highly sensitive functional assessment of immune competence.

Traditionally, TDAR assays have employed sheep red blood cells or KLH in experimental animals. In human studies, immune competence has frequently been evaluated using antibody responses to licensed vaccines, including tetanus, diphtheria, pertussis, influenza, or hepatitis B vaccines. Although these vaccines offer well-established safety profiles and provide direct clinical benefit to study participants, their application is limited by previous vaccination or natural exposure, which may substantially influence immune responses. Furthermore, these highly immunogenic vaccines often induce near-maximal antibody responses, reducing their sensitivity for detecting subtle immunomodulatory effects. In contrast, KLH fulfills many characteristics of an ideal TD antigen by eliciting a reproducible primary immune response in immunologically naïve individuals, thereby allowing standardized assessment of adaptive immune function under controlled experimental conditions [23].

### 6.2. Immunological Mechanisms and Assessment of KLH-Induced Responses

KLH immunization provides a well-characterized and robust antigenic stimulus for the assessment of multiple components of the adaptive immune response within a single experimental model. Following intramuscular, subcutaneous, or intradermal administration, conventional parameters of antigen-specific immunity can be evaluated using complementary immunological approaches, including measurements of KLH-specific IgM, IgG, and IgA antibodies, lymphocyte proliferation, cytokine secretion, enzyme-linked immunosorbent spot (ELISpot) assays for antibody-secreting cells, flow cytometric characterization of antigen-specific lymphocyte populations, and DTH responses following intradermal antigen challenge. Importantly, these readouts reflect the coordinated cellular and humoral processes that characterize adaptive immune responses to protein antigens in general, rather than mechanisms unique to KLH. The particular value of KLH therefore lies in its strong and reproducible immunogenicity, well-established use as a neoantigen, and suitability for standardized assessment of these responses across different experimental and clinical settings. Nevertheless, considerable variability in assay design, immunization protocols, and outcome measures remains evident across studies, highlighting the need for greater methodological standardization [12].

Among the in vivo approaches, DTH testing represents a robust functional measure of antigen-specific cellular immunity. Following prior immunization, intradermal KLH challenge induces localized T-cell-mediated inflammation characterized by infiltration of activated lymphocytes and inflammatory cells. Randomized clinical studies have demonstrated that these responses are highly reproducible and sufficiently sensitive for evaluating pharmacological modulation of cellular immunity [43].

Beyond conventional serological analyses, advances in immunomonitoring have enabled detailed characterization of antigen-specific immune responses. Flow cytometric studies have demonstrated that frequency of KLH-specific B cells strongly correlates with serum anti-KLH antibody concentrations, providing a complementary measure of the magnitude and maturation of the humoral response. Repeated KLH immunization can consequently be used to monitor the transition from naive B-cell populations toward class-switched and memory B-cell populations, representing canonical stages of antigen-specific B-cell maturation [44]. Similarly, combining multiplex cytokine assays with KLH-specific B-cell ELISpot analysis has provided comprehensive evaluation of humoral immunity in both healthy individuals and patients with systemic lupus erythematosus receiving low-dose immunosuppressive therapy. The preservation of primary and secondary IgG responses, predominantly of the IgG1 subclass, confirmed that KLH remains an effective model for assessing T-cell-dependent humoral immunity under diverse clinical conditions [45].

Collectively, these findings support the use of KLH as a practical and reproducible model for monitoring established components of adaptive immunity, including antigen-specific antibody production, B- and T-cell activation, cytokine responses, and the development of immunological memory. Rather than inducing immune processes that are fundamentally distinct from those elicited by other protein antigens, KLH is generally considered a neoantigen, with limited pre-existing KLH-specific immunity expected in the general population, and offers an experimentally convenient and well-characterized antigenic system in which these processes can be readily quantified [46].

### 6.3. Determinants of KLH Immunogenicity

The magnitude and quality of KLH-induced immune responses are influenced by multiple experimental and host-related factors. KLH has been successfully administered through several routes, including intramuscular, subcutaneous, intradermal, and inhalational delivery. Intramuscular administration into the deltoid muscle represents the most frequently employed immunization strategy, whereas intradermal administration is commonly used for subsequent DTH challenge and assessment of local cellular immunity.

Clinical studies have employed KLH doses ranging from 8 µg to 5000 µg, although doses between 100 and 1000 µg are most commonly used. Interestingly, dose-escalation studies demonstrated that high molecular weight (HMW) KLH induced comparable antibody kinetics and response magnitudes across doses ranging from 10 µg to 5000 µg, indicating exceptionally high intrinsic immunogenicity even at relatively low antigen concentrations [46]. Nevertheless, substantial interindividual variability remains evident, with age, physical activity, psychological stress, chronic disease, and immunosuppressive treatment all influencing both humoral and cellular responses. Reduced responsiveness has been consistently observed among immunocompromised individuals, whereas healthy and physically active subjects generally develop stronger adaptive immune responses [47,48].

KLH intended for clinical application is available as either native HMW-KLH or purified subunit KLH. HMW-KLH retains its native multimeric structure and consistently exhibits superior immunogenicity, whereas subunit KLH demonstrates reduced immunostimulatory capacity unless administered together with an adjuvant. Comparative clinical studies showed that immune responses induced by HMW-KLH were comparable to those achieved with adjuvanted subunit KLH formulated with Montanide ISA 51, while non-adjuvanted subunit KLH elicited substantially weaker responses [48]. These observations suggest that the enhanced immunogenicity of HMW-KLH primarily results from its native structural organization and intrinsic immunostimulatory properties rather than differences in antigenic epitopes alone.

### 6.4. Safety and Tolerability of KLH

KLH has generally been well tolerated in clinical studies, although local reactogenicity has been reported and appears to depend on dose, formulation, route of administration, and possibly adjuvant. Notably, a systematic review identified 45 human KLH challenge studies, with HMW-KLH used in 30 and subunit KLH in 14 studies. In one study using a 5 mg subcutaneous dose, approximately 38% of participants withdrew because of large local reactions, highlighting the importance of dose and formulation when interpreting the safety profile of KLH [46]. Other findings demonstrated that repeated low-dose oral KLH can modulate a pre-existing KLH-specific immune response. Following parenteral KLH immunization, volunteers received low-dose oral KLH (5 mg daily for 10 days), which, despite having no significant effect on KLH-specific CD4^+^ T-cell proliferation, DTH responses, or serum levels of KLH-specific IgA, IgM, IgG, and IgG subclasses, markedly altered the functional phenotype of KLH-specific T cells [49,50].

Across numerous human studies, no serious adverse events have been reported. Adverse reactions are generally limited to mild transient local injection-site reactions, although caution is recommended in individuals with shellfish allergy because of potential allergic reactions. The incidence of local reactogenicity may also vary according to the adjuvant formulation employed [12,51].

### 6.5. Clinical Applications of the KLH Challenge Model

The KLH challenge model has become an established translational platform for evaluating pharmacodynamic effects and proof-of-mechanism during early clinical development of immunomodulatory therapies. Standard protocols typically involve primary intramuscular or intradermal immunization followed by booster immunizations and subsequent intradermal antigen challenge, enabling simultaneous assessment of systemic antibody responses and local recall immunity [52].

Comprehensive analyses of multiple clinical trials have demonstrated that repeated KLH immunization significantly enhances both systemic anti-KLH antibody production and local skin inflammation following intradermal challenge, with three immunizations producing the most robust and reproducible responses. Quantitative skin imaging performed approximately 24 h after antigen challenge has been identified as the optimal time point for assessing local immune activation, while significant correlations between systemic humoral responses and local inflammatory reactions further support the biological consistency of the model [47].

The translational value of the KLH challenge model has been further demonstrated in studies evaluating novel immunomodulatory therapies. Repeated KLH immunization has proven sufficiently sensitive to detect dose-dependent suppression of both humoral and cellular immune responses following treatment with the anti-OX40L monoclonal antibody amlitelimab, thereby supporting its application for mechanism-of-action studies and dose selection during early clinical development [43]. In addition, systematic evaluation of clinical studies conducted between 1994 and 2022 concluded that KLH-based immune challenge models provide valuable information regarding biological activity, target engagement, and pharmacodynamic effects of investigational therapies. Nevertheless, substantial methodological heterogeneity remains among published studies, emphasizing the need for standardized protocols and more comprehensive immunophenotyping to maximize translational applicability [12].

Beyond serving as a general model antigen, KLH has also been explored as a carrier for inducing immune responses against tumor-associated carbohydrate antigens (TACAs). Carbohydrate microarray analyses demonstrated that KLH formulated with alum can induce measurable anti-carbohydrate antibody responses in a subset of individuals, although considerable interindividual variability was observed. Baseline antibody repertoires appeared to influence vaccine responsiveness, suggesting that individual immune background may represent an important determinant of adaptive responses elicited by KLH-based immunotherapies [53].

Overall, KLH represents one of the most versatile model antigens currently available for investigating adaptive immunity in humans. Its ability to induce reproducible primary TD immune responses, combined with excellent safety, broad applicability across diverse immunological assays, and high sensitivity to pharmacological modulation, makes KLH an invaluable translational tool for immunotoxicology, vaccine development, and early-phase clinical evaluation of immunomodulatory therapies.

## 7. Established Carrier Proteins for Glycoconjugate Vaccines

The introduction of glycoconjugate vaccines has fundamentally transformed the prevention of diseases caused by encapsulated bacterial pathogens. Capsular polysaccharides are typically T-cell-independent type 2 antigens that predominantly stimulate marginal zone B cells, resulting in limited immunogenicity in infants younger than two years of age, poor affinity maturation, restricted immunological memory formation, and weak booster responses upon re-exposure [54,55]. Covalent conjugation of polysaccharides to an immunogenic carrier protein overcomes these limitations by enabling antigen processing and presentation through MHC II molecules, thereby recruiting CD4^+^ T helper cells and inducing a T-cell-dependent immune response. These carrier molecules are xenobiotic proteins, including, in some cases detoxified bacterial toxins. Owing to the foreign origin and structural heterogeneity, they efficiently stimulate immune response in humans. Their high molecular weight promotes the uptake of short peptide antigens by antigen-presenting cells (APCs) through enhanced phagocytic processes and can reduce the rate of peptide clearance from the injection site [56].

Immunogenicity of glycoconjugate vaccines is determined not only by the antigenic polysaccharide itself but also by its structural characteristics and conjugation strategy. Specifically, both the polysaccharide chain length and the mode of attachment to the carrier protein play critical roles in shaping the immune response. Cross-linked conjugates containing longer polysaccharide chains generally exhibit enhanced immunogenicity, whereas end-linked conjugates achieve comparable immune responses with intermediate-length oligosaccharides [57]. These findings indicate that there is no universally optimal glycan chain length; instead, vaccine efficacy depends on the combined effects of glycan size and conjugation chemistry.

Since the first successful implementation of glycoconjugate technology in *Haemophilus influenzae* type B (HiB) vaccines during the 1980s, carrier proteins have become a critical component of vaccine design [58]. The choice of carrier influences not only the magnitude and quality of the anti-polysaccharide response but also manufacturing feasibility, conjugation efficiency, physicochemical stability, and the risk of carrier-induced epitope suppression (CIES) [59]. Several carrier proteins have been incorporated into licensed vaccines, including tetanus toxoid (TT), diphtheria toxoid (DT), cross-reacting material 197 (CRM_197_), *Haemophilus influenzae* protein D (HiD), and the outer membrane protein complex (OMPC). Their principal characteristics, limitations, and representative vaccine applications are summarized in Table 1.

TT remains one of the most extensively studied and clinically validated carrier proteins. Native tetanus neurotoxin produced by *Clostridium tetani* is a 150 kDa protein consisting of a 100 kDa heavy chain and a 50 kDa light chain linked by a disulfide bond. Detoxification is achieved through prolonged formaldehyde treatment, resulting in the formation of tetanus toxoid while preserving the majority of immunologically relevant epitopes [61]. The large molecular size of TT provides a high density of lysine residues and numerous T helper epitopes suitable for conjugation. TT has been successfully employed in licensed vaccines including MenAfriVac^®^, Nimenrix^®^, ActHIB^®^, and several investigational pneumococcal and typhoid conjugate vaccines [55,62]. An important feature of TT is its ability to simultaneously boost pre-existing anti-tetanus immunity, which may represent an additional public health benefit in regions with suboptimal vaccination coverage [60]. Nevertheless, formaldehyde detoxification introduces significant molecular heterogeneity. Covalent cross-linking between lysine, tyrosine, histidine, and arginine residues can generate structurally heterogeneous populations that vary between manufacturing batches [61]. Such modifications may alter the accessibility of conjugation sites and complicate physicochemical characterization. Furthermore, repeated exposure to TT through routine childhood immunization schedules can contribute to CIES, particularly when multiple TT-conjugated vaccines are administered within a relatively short time interval [59,60].

DT was among the earliest carrier proteins used in glycoconjugate vaccine development and remains a component of several licensed products. Similar to TT, DT is produced through formaldehyde detoxification of the native diphtheria toxin secreted by toxigenic strains of *Corynebacterium diphtheriae*. The native toxin is a 58 kDa single-chain protein consisting of catalytic, translocation, and receptor-binding domains [61,63]. Historically, DT played a pivotal role in the development of early HiB conjugate vaccines, demonstrating that protein conjugation could successfully convert polysaccharide antigens into T-dependent immunogens [58]. DT contains numerous T helper epitopes and efficiently induces helper responses necessary for anti-polysaccharide antibody production. In addition, vaccination may contribute to maintenance of protective anti-diphtheria antibody titers [53]. Despite its successful history, DT has gradually been replaced by CRM_197_ in many vaccine formulations. Formaldehyde treatment alters lysine residues and generates intermolecular cross-links, resulting in reduced structural homogeneity compared with CRM_197_. This heterogeneity can affect conjugation reproducibility and analytical characterization. Moreover, CRM_197_ generally provides equivalent immunogenicity while offering substantial manufacturing and regulatory advantages [61,64,65].

CRM_197_ is currently the dominant carrier protein in modern glycoconjugate vaccine development and is incorporated into numerous licensed vaccines, including Prevenar 13^®^, Vaxneuvance^®^, Menveo^®^, Menjugate^®^, and several investigational conjugate vaccines [64]. CRM_197_ originates from a naturally occurring mutant strain of *Corynebacterium diphtheriae* (strain C7(β)197) carrying a single Gly52→Glu substitution within the catalytic domain of diphtheria toxin. This mutation abolishes ADP-ribosyltransferase activity while preserving the native tertiary structure and immunological properties of the toxin [64,66]. Structurally, CRM_197_ is a 58 kDa monomeric protein composed of 535 amino acids and contains approximately 39 lysine residues available for polysaccharide conjugation [64]. The absence of chemical detoxification is one of its most important advantages. In contrast to DT, CRM_197_ maintains a highly defined molecular structure with minimal batch-to-batch variability, facilitating analytical characterization and regulatory approval. Several studies have demonstrated that CRM_197_ contains multiple promiscuous CD4+ T-cell epitopes capable of binding diverse human leukocyte antigen (HLA) class II molecules, which contributes to its broad immunogenicity across genetically diverse populations [59,65]. The widespread use of CRM_197_ has, however, raised concerns regarding carrier-induced immune interference. Studies evaluating sequential administration of CRM_197_-containing vaccines have reported evidence of carrier-specific memory responses competing with polysaccharide-specific B-cell activation. Although the clinical significance of this phenomenon remains debated, it has stimulated interest in diversifying carrier protein selection for future glycoconjugate vaccines [67].

Protein D (PD) represents a fundamentally different carrier concept because it functions not only as a carrier protein but also as a vaccine antigen. It is a highly conserved 42 kDa surface lipoprotein expressed by more than 80% of non-typeable *Haemophilus influenzae* (NTHi) strains. The protein is involved in glycerophosphodiester phosphodiesterase activity and contributes to bacterial adherence and colonization of the respiratory tract [68,69]. PD was selected as the carrier protein for ten of the thirteen pneumococcal serotypes included in Synflorix^®^ (PCV10) [68]. The rationale was that vaccination might simultaneously induce antibodies against pneumococcal capsular polysaccharides and NTHi surface antigens. Clinical studies indeed demonstrated robust anti-protein D antibody responses following immunization, although the degree of protection against NTHi-associated diseases remains a subject of ongoing investigation [68]. Compared with TT and CRM_197_, Protein D offers the advantage of limited pre-existing immunity in vaccinated populations and reduced risk of carrier-induced suppression. However, its use remains largely restricted to Synflorix^®^, resulting in a comparatively limited clinical evidence base [55]. In addition, the protein possesses fewer established T-cell epitopes than classical toxoid carriers, which may influence carrier performance depending on the conjugated antigen and target population.

OMPC differs substantially from other licensed carrier proteins because it is not a single purified protein but rather a heterogeneous complex derived from the outer membrane of *Neisseria meningitidis* serogroup B. The complex contains multiple outer membrane proteins, phospholipids, and lipooligosaccharide-associated components that collectively contribute to its immunostimulatory activity [60,70]. OMPC gained prominence through its incorporation into PedvaxHIB^®^, one of the first successful HiB conjugate vaccines. Notably, OMPC-conjugated HiB vaccines elicited protective antibody concentrations after fewer doses than several alternative HiB formulations, particularly in very young infants [71]. This enhanced immunogenicity is believed to result from the intrinsic adjuvant-like properties of meningococcal membrane components, which activate innate immune pathways and promote efficient antigen presentation. The principal disadvantage of OMPC lies in its complexity. Unlike CRM_197_ or PD, OMPC cannot be fully described as a single molecular entity. Batch characterization, quality control, and regulatory assessment are therefore inherently more challenging. These considerations, combined with advances in recombinant protein technologies, have limited its incorporation into newer glycoconjugate vaccine platforms [55,72].

Although licensed glycoconjugate vaccines continue to rely predominantly on CRM_197_, TT, DT, PD, and OMPC, increasing attention has focused on alternative carrier proteins capable of mitigating carrier-induced immune interference and expanding available T-cell epitope repertoires. Among the most extensively investigated candidates is KLH. The exceptional immunogenicity of KLH is largely attributed to its phylogenetic distance from mammalian proteins, structural complexity, and repetitive epitope organization. Consequently, KLH has become one of the most widely used carrier proteins in experimental immunology, therapeutic cancer vaccines, anti-drug antibody generation, and hapten immunization studies. Several clinical trials have demonstrated its capacity to induce strong antigen-specific humoral and cellular immune responses without requiring additional carrier-specific priming. Despite these promising characteristics, KLH has not yet been incorporated into licensed glycoconjugate vaccines. Major challenges include its intrinsic molecular heterogeneity, natural-source production, complex purification procedures, and difficulties in achieving the level of structural characterization currently expected for licensed biopharmaceutical products. Nevertheless, advances in analytical methods, structural biology, and bioprocess development continue to increase interest in KLH as a next-generation carrier protein for glycoconjugate vaccine applications. However, all of these facts are accompanied with the higher cost necessary for the research and development of such type of vaccine, which logically will result in a higher price of the final drug product (vaccine) used in the therapeutic practice. Another very important factor which can significantly affect the price is the availability of the KLH. A significant issue is that the KLH protein is obtained from a natural source (gastropod mollusk *M. crenulata*) with a limited amount. However, that mollusk can be maintained in captivity without negatively affecting the relative levels of KLH isoforms, and so its reliable long-term supply can be provided through aquaculture [73,74].

On the other side, large variety of studies identified some specific factors, such as overfishing [74], marine heat waves [75], increase in temperature in the Northeastern Pacific Ocean [76,77,78], which have led to a decline in the population of marine mollusks, including *Megathura crenulata*. Therefore, if such phenomena continue to occur, local extinction of these marine mollusks may eventually occur. These concerns regarding the KLH source security at appropriate level may be the main reason why KLH still remains as an unestablished carrier protein by the regulatory agencies.

## 8. KLH in Vaccinology and Immunotherapy

### 8.1. Alzheimer’s Disease

Alzheimer’s disease (AD) is the most common neurodegenerative disorder and the leading cause of dementia worldwide, characterized by progressive cognitive decline, memory impairment, and irreversible neuronal loss. The pathophysiology of AD is complex and multifactorial, with the two principal neuropathological hallmarks being the extracellular accumulation of amyloid-β (Aβ) plaques and the intracellular formation of neurofibrillary tangles composed of hyperphosphorylated tau protein [79]. According to the amyloid cascade hypothesis, abnormal cleavage of amyloid precursor protein (APP) by β- and γ-secretases leads to excessive production and aggregation of Aβ peptides, particularly Aβ42, which exert neurotoxic effects, promote oxidative stress, synaptic dysfunction, and trigger chronic neuroinflammation mediated by activated microglia and astrocytes. Concurrently, tau hyperphosphorylation destabilizes neuronal microtubules, resulting in impaired axonal transport and progressive neuronal degeneration. Increasing evidence further suggests a synergistic interplay between Aβ pathology, tau propagation, mitochondrial dysfunction, and neuroinflammatory processes in disease progression.

Nowadays, therapeutic strategies are largely focused on symptomatic treatment using acetylcholinesterase inhibitors and N-methyl-D-aspartate (NMDA) receptor antagonists; however, recent advances increasingly emphasize disease-modifying approaches, particularly monoclonal antibodies and immunotherapies targeting Aβ and tau aggregates. Despite partial clinical success of anti-amyloid antibodies such as lecanemab and donanemab, their efficacy remains limited and associated with safety concerns, highlighting the need for alternative and more effective therapeutic modalities [80]. Active immunization strategies and therapeutic vaccines have emerged as promising approaches aimed at inducing long-term immune responses against pathological protein aggregates. In this context, carrier proteins such as KLH are being extensively investigated as potent immunogenic platforms capable of enhancing antibody production against weakly immunogenic Aβ or tau epitopes, thereby representing an important direction in the development of next-generation Alzheimer’s disease vaccines and immunotherapeutics.

Zhang et al. investigated the therapeutic potential of the active immunization vaccine Aβ3–10-KLH in APP/PS1 transgenic mice, a widely used murine model of AD. The vaccine consists of the N-terminal Aβ3–10 epitope conjugated to KLH, which serves as an immunogenic carrier protein. The rationale behind this construct was to preserve the B-cell epitope capable of inducing anti-Aβ antibody production while excluding the C-terminal Aβ regions associated with T-cell-mediated autoimmune responses observed in earlier AD vaccine trials. Six-month-old APP/PS1 mice were immunized six times at two-week intervals, resulting in a robust humoral immune response characterized by high serum anti-Aβ antibody titers. Vaccinated animals demonstrated significant improvements in spatial learning and memory in the Morris water maze test, indicating that the vaccine was capable not only of inducing immunogenicity but also of ameliorating cognitive deficits associated with AD pathology. Collectively, the study demonstrated that KLH-based active immunization can exert both anti-amyloid and neuroprotective effects, including mitigation of mitochondrial dysfunction and oxidative stress [81].

Park et al. evaluated the immunotherapeutic potential of novel Aβ1–10 peptide epitope vaccines conjugated to carrier proteins, particularly KLH and ovalbumin (OVA), in a murine model of AD. The authors designed three Aβ1–10-derived peptide epitopes (Aβ1–10-N, Aβ1–10-D1H, and Aβ1–10-S8R) using in silico B-cell epitope prediction and molecular docking analyses to optimize antigenicity while minimizing the risk of T-cell-mediated adverse immune reactions. The S8R-modified epitope demonstrated the highest predicted immunogenicity and was subsequently conjugated to KLH or OVA to enhance humoral immune responses. In vivo experiments demonstrated that KLH- and OVA-conjugated vaccines elicited markedly stronger humoral immune responses than unconjugated peptides, as evidenced by significantly elevated splenic and plasma IgG levels. The carrier-conjugated Aβ1–10-S8R vaccines also improved behavioral outcomes in Y-maze and passive avoidance tests, indicating amelioration of Aβ-induced cognitive deficits. Importantly, the KLH-conjugated formulation produced substantial reductions in cerebral Aβ accumulation, amyloid precursor protein (APP), and β-secretase 1 (BACE-1) expression within the cortex and hippocampus. These findings suggest that the vaccine not only promoted Aβ clearance but may also have attenuated amyloidogenic APP processing. The study further demonstrated significant immunomodulatory and neuroprotective effects of the KLH-based vaccine. Vaccination reduced astrocytic and microglial activation, reflected by decreased glial fibrillary acidic protein (GFAP) and ionized calcium binding adaptor molecule 1 (Iba-1) expression, and suppressed pro-inflammatory cytokines including tumor necrosis factor α (TNF-α) and interleukin-1β (IL-1β). In parallel, synaptic integrity was preserved, as shown by increased expression of synaptophysin, SNAP-23, and PSD-95 in vaccinated animals [82]. These findings support the use of KLH as an effective carrier platform in peptide vaccine design and further highlight the therapeutic potential of KLH-conjugated vaccines in neurodegenerative disease immunotherapy.

A different immunotherapeutic direction is represented by tau-targeting vaccines, which aim to reduce pathological tau aggregation rather than extracellular amyloid pathology. The phase I clinical trial of the first tau vaccine AADvac1 tested on humans evaluated safety, tolerability, and immunogenicity in patients with mild-to-moderate Alzheimer’s dementia. The vaccine was designed to induce antibodies against pathological tau species while limiting immune responses against physiological tau. The study demonstrated that active tau immunization was feasible and capable of inducing an antibody response, providing clinical proof-of-concept for targeting tau pathology. However, subsequent development highlighted challenges including the complexity of tau-related disease mechanisms, variability of immune responses among patients, and the need to demonstrate clear clinical efficacy [83,84].

According to the promising results with the specific vaccines focused against tau protein, it can be expected, that further investigation will be focused on improved clinical investigation in this area. KLH as a carrier protein will play a significant role in that investigations what will bring new possibilities to improve its characterization and demands on its quality control. On the other side, the difficulties accompanied with KLH as carrier protein can be solved by its change to more convenient, well-established and approved carriers, which were mentioned and summarized in Table 1.

### 8.2. Atherosclerosis

Atherosclerosis is a chronic progressive inflammatory disease of the arterial wall and the principal underlying cause of cardiovascular disorders such as myocardial infarction, ischemic stroke, and peripheral artery disease [85]. The disease is initiated by endothelial dysfunction and the subendothelial accumulation of low-density lipoproteins (LDLs), which undergo oxidative modification within the vascular intima. Oxidized LDL triggers recruitment of monocytes, activation of endothelial cells, and differentiation of macrophages into lipid-laden foam cells, leading to the formation of atherosclerotic plaques [86]. In parallel, both innate and adaptive immune responses critically contribute to lesion progression through chronic vascular inflammation mediated by macrophages, T lymphocytes, cytokines, and autoantigen-driven immune mechanisms [87]. As plaques evolve, sustained inflammation promotes smooth muscle cell migration, extracellular matrix remodeling, necrotic core formation, and plaque instability, ultimately increasing the risk of thrombosis and acute cardiovascular events [88].

Lipid-lowering agents, particularly statins and proprotein convertase subtilisin/kexin type 9 (PCSK9) inhibitors, combined with antihypertensive and antithrombotic drugs are all therapeutics used in the management of atherosclerosis [89]. However, residual inflammatory risk frequently persists despite optimal lipid control [90,91]. Consequently, recent research has increasingly focused on immunomodulatory and anti-inflammatory approaches targeting cytokine signaling, immune checkpoints, and antigen-specific adaptive immunity [87]. Emerging evidence indicates that vaccination strategies directed against atherosclerosis-associated antigens, including oxidized LDL, apolipoprotein B-100, heat shock proteins, and PCSK9, may reduce vascular inflammation and attenuate plaque development [92,93]. In this context, KLH has attracted attention as a highly immunogenic carrier protein capable of enhancing immune responses against weakly immunogenic atherosclerosis-related epitopes. KLH-conjugated vaccine platforms are therefore being explored as promising tools for the development of antigen-specific immunotherapies aimed at long-term prevention and modulation of atherosclerotic disease progression [12].

Me et al. investigated a novel immunotherapeutic strategy targeting vascular remodeling and atherosclerosis. A disintegrin and metalloproteinase with thrombospondin motifs-7 (ADAMTS-7) has previously been identified as a key mediator of vascular smooth muscle cell (VSMC) migration and extracellular matrix degradation, processes that are central to both atherosclerotic plaque development and restenosis following vascular injury. The authors developed a peptide-based vaccine designed to induce an immune response against ADAMTS-7. Using experimental murine models of atherosclerosis and vascular injury, they demonstrated that immunization significantly reduced atherosclerotic plaque burden and inhibited neointimal hyperplasia after arterial injury. Mechanistically, the vaccine elicited a specific antibody response that suppressed ADAMTS-7 activity, thereby limiting VSMC migration and proliferation—two critical steps in pathological vascular remodeling. Importantly, the intervention was associated with reduced inflammatory signaling within the vascular wall, suggesting that modulation of immune responses against key extracellular proteases can influence both structural and inflammatory components of atherosclerosis [94].

Overall, this study provides proof-of-concept evidence that vaccination strategies targeting disease-associated proteins such as ADAMTS-7 may represent a novel and potentially long-lasting therapeutic approach for the prevention of atherosclerosis progression and post-interventional restenosis. These results support the broader concept of immunomodulation as an emerging avenue in cardiovascular disease management, complementing traditional lipid-lowering and anti-inflammatory therapies.

### 8.3. Substance Use Disorder

Substance use disorder (SUD) represents a chronic relapsing neuropsychiatric condition characterized by compulsive drug seeking and consumption despite harmful physiological, psychological, and social consequences. The disorder develops through complex interactions between addictive substances and neural circuits involved in reward processing, motivation, learning, and executive control [95]. Repeated exposure to drugs such as opioids, nicotine, cocaine, methamphetamine, or fentanyl induces profound neuroadaptive changes within the mesocorticolimbic dopaminergic system, particularly in the ventral tegmental area, nucleus accumbens, amygdala, and prefrontal cortex, which regulate reward, motivation, and executive control [96]. These alterations involve glutamatergic dysregulation, long-term synaptic plasticity, stress-related neurocircuitry activation, and maladaptive memory formation, ultimately leading to tolerance, dependence, craving, and relapse [97]. Chronic substance exposure is additionally associated with neuroinflammation, oxidative stress, epigenetic modifications, and long-term synaptic remodeling that contribute to the persistence and recurrence of addictive behaviors even after prolonged abstinence [98,99].

Current therapeutic approaches include behavioral interventions, cognitive and motivational therapies, and pharmacological treatments such as methadone, buprenorphine, naltrexone, nicotine replacement therapy, or varenicline. Treatment efficacy remains limited by high relapse rates, poor adherence, and the absence of effective medications for several classes of abused substances [100,101].

Contemporary research increasingly focuses on biologically targeted interventions capable of preventing drug distribution to the central nervous system. Among these strategies, anti-drug vaccines have emerged as a promising immunopharmacological approach designed to elicit the production of high-affinity antibodies that bind drug molecules in the bloodstream and reduce their penetration across the blood–brain barrier [102,103]. Because most addictive substances are small haptens with inherently low immunogenicity, they are commonly conjugated to highly immunostimulatory carrier proteins, particularly KLH, which substantially enhances antigen presentation and antibody generation [9,46]. KLH-based conjugate vaccines are therefore being actively investigated in experimental and clinical studies targeting nicotine, cocaine, opioids, and synthetic drugs as potential long-term therapeutic tools for the prevention and management of SUD.

Drug pharmacodynamics and pharmacokinetics are critical factors in determining the effectiveness of vaccines against substance use disorders. Highly potent drugs, such as fentanyl, are generally more suitable vaccine targets because antibody binding can produce substantial shifts in the dose–response relationship, whereas less potent drugs require considerably higher antibody concentrations to achieve comparable effects. Hapten design must also account for drug metabolism, as vaccines should target the pharmacologically active compound, whether it is the parent drug (e.g., cocaine) or an active metabolite (e.g., 6-acetylmorphine derived from heroin). Antibody binding prolongs the drug’s half-life by reducing its metabolism and clearance, with the magnitude of this effect varying among drugs according to their metabolic pathways. Although an extended half-life can enhance peripheral drug sequestration, excessively prolonged antibody–drug complexes may become saturated during repeated drug exposure, potentially limiting vaccine efficacy [15].

Walter et al. investigated the in vivo safety of a nanoparticle-based vaccine platform designed for opioid use disorder (OUD), using subunit KLH encapsulated in lipid–poly(lactic-co-glycolic) acid (PLGA) hybrid nanoparticles (sKLH-hNPs) in BALB/c mice. Mice received subcutaneous injections of either a low (60 μg) or high (300 μg) dose and were monitored for up to 56 days. Safety was assessed through behavioral observations, serum biochemical parameters (including liver, kidney, electrolyte, and metabolic markers), and comprehensive histopathological analysis of major organs and injection sites. The results showed no meaningful dose-dependent toxicity or systemic adverse effects, with normal metabolic and organ function across all groups. Histological evaluation revealed only minor and nonspecific tissue changes that were also present in control animals. Behavioral assessments were largely unaltered, with only a transient difference in respiration observed on day 3 [104]. The trend of lipid-based nanocarrier delivery systems is further supported also by another studies, where liposomes have been used as carriers for heroin and nicotine haptens [105,106].

Another study by Zhao et al. was primarily focused on a KLH-conjugated nanoparticle-based nicotine vaccine designed to enhance the immunogenicity and therapeutic efficacy of anti-nicotine immunotherapy. The authors evaluated how nanoparticle size influences immune activation, antibody production, and vaccine performance. By coupling nicotine antigens to KLH, the vaccine exploited the strong carrier and immunostimulatory properties of KLH to induce robust nicotine-specific antibody responses. The findings demonstrated that nanoparticle formulation and particle size significantly affected immunogenicity, with optimized nanoparticles improving antibody titers and functional sequestration of nicotine [107].

Prescription opioids represent an important target for vaccine-based immunotherapy, as their widespread medical use has contributed to a significant increase in opioid misuse, dependence, and overdose. Therefore, the application of conjugate vaccine strategies is not limited only to illicitly used substances such as heroin or cocaine but also extends to commonly prescribed opioids with high abuse potential. Kimishima et al. demonstrated the potential of opioid vaccines by developing a hapten–carrier protein conjugate vaccine capable of inducing high-affinity antibodies against prescription opioids. The generated antibodies sequestered opioids in the bloodstream, significantly altered their pharmacokinetic profile by increasing drug half-life, reduced central nervous system exposure, attenuated opioid-induced effects, and provided protection against overdose in animal models [108]. These findings provided important preclinical evidence supporting the translation of opioid vaccines into clinical studies. Building on this approach, the present clinical study (estimated study completion: March 2027) is designed to investigate a novel therapeutic strategy for OUD, specifically the use of a vaccine directed against oxycodone, Oxy(Gly)4-sKLH, which targets one of the most frequently misused prescription opioids. Vaccine-based treatment for substance use disorders is based on the induction of drug-specific antibodies that bind the target opioid in the bloodstream and limit its passage across the blood–brain barrier, thereby reducing its psychoactive effects. The broader objective of this research program is the future development of a combined vaccine targeting both oxycodone and heroin; however, the current trial evaluates the Oxy(Gly)4-sKLH vaccine as a standalone intervention. The study is being conducted at multiple sites, including the New York State Psychiatric Institute and the Clinilabs Clinical Research Unit in Eatontown, New Jersey. Its primary aims are to assess safety, degree of antibody production and efficacy [109].

## 9. KLH in Cancer Immunotherapy

The antitumor activity of KLH is primarily associated with its potent immunostimulatory properties and its ability to activate both innate and adaptive immune responses. As a highly immunogenic xenogeneic glycoprotein, KLH promotes antigen uptake and maturation of dendritic cells, enhances antigen presentation through MHC pathways, and stimulates robust CD4+ T-cell, cytotoxic T-cell, and B-cell responses accompanied by cytokine production, including IL-2, IFN-γ, TNF-α, and other pro-inflammatory mediators. In addition to functioning as a nonspecific immune activator, KLH acts as an effective carrier protein for poorly immunogenic tumor-associated antigens, thereby amplifying tumor-specific immune recognition and antibody generation. These mechanisms have led to extensive investigation of KLH in cancer vaccine development and tumor immunotherapy, particularly in strategies targeting immune priming and long-term immunological memory [110,111]. Current trends in cancer immunotherapy increasingly focus on personalized neoantigen vaccines, dendritic-cell-based platforms, combination therapies with immune checkpoint inhibitors, and conjugate vaccines designed to overcome tumor immune evasion and low antigen immunogenicity [112,113]. Within this context, KLH remains one of the most widely utilized carrier proteins because of its excellent safety profile and strong capacity to enhance T-cell-dependent immune responses. Experimental and clinical studies have demonstrated promising applications of KLH-based immunotherapy in bladder cancer, where intravesical or systemic KLH administration exhibited significant antitumor effects and reduced tumor recurrence, supporting its role as a potential alternative or adjunct to Bacillus Calmette–Guérin (BCG) therapy [114,115]. Furthermore, KLH-conjugated vaccines targeting mucin-like antigens, gangliosides, human epidermal growth factor receptor 2 (HER2)-related epitopes, and other tumor-associated molecules have been investigated in breast cancer, melanoma, pancreatic cancer, prostate cancer, ovarian carcinoma, and colorectal malignancies, where KLH serves primarily as an immunogenic carrier capable of potentiating otherwise weak anticancer immune responses [51,116]. Ongoing research is now directed toward optimizing KLH-based vaccine formulations through novel adjuvants, nanoparticle delivery systems, multi-epitope antigen constructs, and combination regimens with checkpoint blockade therapies in order to achieve more durable and tumor-specific immune activation while minimizing systemic toxicity [113].

### 9.1. Bladder Cancer

KLH has also been applied clinically as an immunotherapeutic agent for the prevention of bladder cancer recurrence following surgical intervention. Although therapeutic responses can be substantial in some patients, clinical benefit is observed only in a subset of individuals, highlighting significant interpatient variability. There is a need to optimize its clinical application by developing predictive strategies to identify patients most likely to respond, as well as by establishing reliable biomarkers that can indicate, ideally at early stages of treatment, whether a favorable immune response is being achieved [114,117]. Furthermore, elucidating the mechanisms underlying KLH-mediated antitumor activity remains an important objective, as this knowledge would not only advance fundamental understanding of tumor immunology but also support biomarker discovery and improve the rational design of KLH-based immunotherapeutic approaches.

Oyelaran et al. used carbohydrate microarray to profile the repertoire of antibody responses induced by KLH with alum adjuvant in human subjects. Their result provided the most comprehensive evaluation of anti-carbohydrate antibody responses published to date and have implications for basic and clinical research on KLH [53]. Lamm et al. tested the antitumor effects of KLH in a mouse model of bladder cancer (MBT2). They administered KLH directly into the tumors (intralesional injection) and monitored its impact on tumor development, growth rate, and survival of the animals. The results were compared to treatment with BCG, a standard immunotherapy for bladder cancer. Additionally, they evaluated whether endotoxin contamination contributed to the observed effects by testing endotoxin separately. The study concludes that purified KLH has significant antitumor activity in the MBT2 mouse model of bladder cancer and shows efficacy comparable to BCG [17].

### 9.2. Breast Cancer

KLH has been extensively explored as a carrier protein in conjugate cancer vaccines targeting antigens including mucin-1 (MUC1), globohexaosylceramide H (Globo H), and sialyl-Tn (STn). Early breast cancer vaccine studies demonstrated that MUC1-KLH formulations combined with adjuvants such as QS-21 were capable of inducing both humoral and cellular antitumor immune responses with acceptable toxicity profiles in patients with advanced disease. More recently, KLH-based vaccine strategies have evolved toward glycan-targeted and personalized immunotherapies. The Globo H-KLH conjugate vaccine adagloxad simolenin (OBI-822) showed the ability to induce specific anti-Globo H antibodies in metastatic breast cancer, supporting the concept that KLH-conjugated carbohydrate vaccines can generate clinically relevant immune activation [16]. Similarly, the STn-KLH vaccine (Theratope^®^) represented one of the largest phase III evaluations of therapeutic vaccination in metastatic breast cancer. Miles et al. evaluated the therapeutic efficacy of Theratope^®^ in 1028 patients with metastatic breast cancer. In this vaccine strategy, the tumor-associated carbohydrate antigen STn was conjugated to KLH to enhance immunogenicity and induce a targeted antitumor humoral immune response. The study assessed clinical endpoints including time to progression and overall survival, while also monitoring vaccine-induced immune responses. Although vaccination elicited a strong and specific antibody response against the STn antigen, the trial did not demonstrate a significant improvement in survival or disease progression in the overall study population. Nevertheless, the study provided important insights into the immunological potential of KLH-conjugated cancer vaccines and highlighted the capacity of KLH to function as a potent carrier protein capable of generating robust antigen-specific immune activation in oncology settings [51].

Current trends in KLH-directed research increasingly focus on optimizing antigen selection, improving vaccine adjuvant systems, combining KLH-based vaccines with immune checkpoint inhibitors, and integrating neoantigen-driven personalized vaccine approaches. Another major direction involves exploiting KLH not only as an immunostimulatory molecule itself but also as a universal carrier for weakly immunogenic tumor antigens in dendritic-cell vaccines and peptide-based platforms. Overall, contemporary research suggests that the future of KLH in breast cancer immunotherapy will likely depend on rational combinatorial strategies, biomarker-guided patient stratification, and integration with modern precision immuno-oncology platforms rather than its use as a standalone vaccine component.

### 9.3. Melanoma

Melanoma is an aggressive malignancy arising from melanocytes and represents the deadliest form of skin cancer despite accounting for only a small proportion of all cutaneous malignancies. Its incidence has steadily increased over recent decades, particularly in fair-skinned populations exposed to ultraviolet radiation [118]. Although early-stage melanoma can often be cured by surgical excision, advanced and metastatic disease is associated with a significantly poorer prognosis due to its high metastatic potential and remarkable biological heterogeneity. Importantly, melanoma is considered one of the most immunogenic human cancers, characterized by a high mutational burden, abundant tumor-associated antigens, and frequent infiltration by immune cells within the tumor microenvironment [119,120]. These features have established melanoma as a prototypical model for the development of cancer immunotherapy, including cytokine-based treatments such as high-dose interleukin-2 (IL-2), immune checkpoint inhibitors targeting cytotoxic T-lymphocyte-associated protein 4 (CTLA-4), adoptive cell therapies, and therapeutic cancer vaccines [121,122,123]. Long before the advent of modern checkpoint blockade, melanoma served as a primary platform for evaluating vaccine-based approaches targeting melanoma-associated antigens, including gangliosides and other tumor-associated carbohydrate antigens conjugated to immunogenic carrier proteins such as KLH [124].

Melanoma was among the first malignancies in which active cancer vaccination strategies based on TACAs were extensively investigated. Melanoma cells frequently overexpress gangliosides such as GM2, GD2, GD3 and GM3 which represent attractive immunotherapeutic targets but exhibit poor intrinsic immunogenicity due to their self-antigen nature [125]. To overcome immune tolerance, synthetic gangliosides were conjugated to KLH with a BCG as an adjuvant [126,127]. This vaccination strategy aimed to induce both humoral and cellular antitumor immunity, resulting in the generation of ganglioside-specific antibodies, activation of T lymphocytes, and antibody-mediated tumor cell killing. The clinical efficacy of KLH-based ganglioside vaccines was investigated in the large randomized phase III trial, which evaluated an adjuvant GM2-KLH vaccine formulation in patients with completely resected stage II melanoma. GM2 ganglioside was conjugated to KLH and formulated with saponin-based adjuvant QS-21 to promote antibody generation and immune activation. A total of 1314 patients were randomly assigned to receive either GM2-KLH/QS-21 vaccination or observation following surgical resection. Although the vaccine induced strong GM2-specific IgM and IgG antibody responses, the trial failed to demonstrate a clinical benefit. After a median follow-up of 4.2 years, GM2-KLH/QS-21 vaccination did not improve relapse-free survival, distant metastasis-free survival, or overall survival compared with observation [128]. Moreover, the intrinsically low immunogenicity of gangliosides often requires repeated administration, elevated antigen concentrations, or strong adjuvant support to achieve effective immune stimulation [129].

Consequently, ganglioside-KLH vaccines were largely superseded by immune checkpoint blockade and personalized neoantigen-based approaches. The experience gained from KLH-based ganglioside vaccines provided critical insights into antigen selection, carrier design, adjuvant requirements, and the importance of coordinating humoral and cellular immunity for effective tumor control. Many of these principles continue to guide the development of next-generation melanoma vaccines, including mRNA-based platforms, dendritic-cell vaccines, and individualized neoantigen-targeted immunotherapies [130,131].

## 10. Conclusions

KLH represents a remarkable marine-derived biomolecule that bridges the fields of structural glycobiology, experimental immunology, and translational medicine. Its distinctive quaternary architecture, extensive glycosylation, and structural divergence from mammalian proteins underlie its strong immunogenicity and have established KLH as a well-characterized model antigen in immunological research. Among its most established applications, KLH is widely used as a neoantigen for the assessment of TDAR and as a tool for evaluating immune competence and treatment-related immunomodulation in preclinical and clinical research.

Beyond these established applications, KLH has also been investigated in clinical studies as an immunological challenge antigen and as a carrier for poorly immunogenic haptens and peptides. Its ability to induce robust and measurable cellular and humoral immune responses has supported its evaluation in vaccine-related approaches and immunotherapeutic strategies, including cancer immunotherapy. However, clinical investigations have produced variable outcomes, and the therapeutic efficacy of KLH-based approaches remains dependent on the disease context, formulation, antigen target, and immunization strategy. Therefore, KLH should be regarded primarily as a clinically investigated immunological platform rather than as an established therapeutic agent.

The KLH molecule as whole remains a significant issue because it is not possible to prepare the molecule in a synthetic way. It is expected that deeper focus will be placed on the individual subunits of the KLH molecule and searching for their specific peptide sequences which will be able to elicit the required immunogenic reaction. These investigations will be accompanied with expansive toxicology and efficacy study.

A broader range of potential applications remains at the preclinical stage. KLH-based conjugates and carrier systems have been explored as experimental strategies for inducing immune responses against targets associated with neurodegenerative and cardiovascular diseases, as well as substance use disorders. These studies highlight the versatility of KLH as an immunogenic carrier and vaccine scaffold, but further evidence is required to determine their clinical relevance and therapeutic efficacy. Future development of KLH-based technologies will also need to address challenges arising from its natural-source origin, molecular heterogeneity, and complex purification and structural characterization. Advances in glycomic and structural analysis, bioprocessing, and formulation technologies may facilitate improved characterization and reproducibility of KLH preparations. Overall, while its role as a model antigen and immunological research tool is well established, the clinical and therapeutic potential of KLH remains an active area of investigation, with preclinical applications providing a basis for the development of future KLH-based vaccines and immunotherapeutic strategies.

## Figures and Tables

**Table 1:** Licensed carrier proteins used in glycoconjugate vaccines: clinical applications examples [55,59,60].

Carrier Protein	M_w_ [kDa]	Origin	Licensed Vaccines	Limitations
TT	~150	*Clostridium tetani* toxoid	ActHIB^®^ MenAfriVac^®^ Nimenrix^TM^ Synflorix^TM^	Pre-existing immunity Risk of CIES
DT	~58–62	*Corynebacterium diphtheriae* toxoid	Menactra^®^	Pre-existing immunity Risk of CIES
CRM_197_	~58	Non-toxic mutant diphtheria toxin	Prevenar13^®^ Prevenar20^®^ Menveo^®^ Vaxneuvance^®^	Pre-existing immunity Risk of CIES
HiD	~40	*Haemophilus influenzae* protein D (outer membrane lipoprotein)	Synflorix^TM^	Limited use outside a single vaccine platform Less extensive clinical experience
OMPC	~100–300	*Neisseria meningitidis* membrane complex	PedvaxHIB^®^ * Comvax^®^	Structural heterogeneity More complex characterization and manufacturing Less extensive clinical experience

* discontinued in 2014.

## Data Availability

No new data were created or analyzed in this study. Data sharing is not applicable to this article.

## Abbreviations

Ab	antibody
AD	Alzheimer’s disease
ADAMTS-7	a disintegrin and metalloproteinase with thrombospondin motifs-7
APCs	antigen-presenting cells
APP	amyloid precursor protein
Aβ	amyloid-β
BACE-1	β-secretase 1
BCG	Bacillus Calmette–Guérin
CIES	carrier-induced epitope suppression
CRM197	cross-reacting material 197
Cryo-EM	cryo-electron microscopy
CTLA-4	T-lymphocyte-associated protein 4
DT	diphtheria toxoid
DTH	delayed-type hypersensitivity
ELISpot	enzyme-linked immunsorbent spot
ER	endoplasmic reticulum
FU	functional unit
Globo H	globohexaosylceramide H
HER2	human epidermal growth factor receptor 2
HiB	*Haemophilus influenzae* type B
HiD	*Haemophilus influenzae* protein D
HLA	human leukocyte antigen class II
HMW	high molecular weight
HPAEC	high-performance anion-exchange chromatography
IL-1β	interleukin-1β
KLH	keyhole limpet hemocyanin
LDLs	low-density lipoproteins
MBT2	model of bladder cancer 2
MHC II	major histocompatibility complex class II
MUC1	mucin-1
NMDA	N-methyl-D-aspartate
NTHi	non-typeable *Haemophilus influenzae*
OMPC	outer membrane protein complex
OUD	opioid use disorder
OVA	ovalbumin
PAGE	polyacrylamide gel electrophoresis
PCKS 9	proprotein convertase subtilisin/kexin type 9
PLGA	poly(lactic-co-glycolic) acid
SDS-PAGE	sodium dodecyl sulfate–polyacrylamide gel electrophoresis
SEC	size exclusion chromatography
sKLH-hNPs	subunit KLH encapsulated in PLGA hybrid nanoparticles
STn	sialyl-Tn
SUD	substance use disorder
TACAs	tumor-associated carbohydrate antigens
TD	T-cell-dependent
TDAR	T-cell-dependent antibody response
TEM	transmission electron microscopy
Tfh	T follicular helper cell
Th	T helper cell
TNF-α	tumor necrosis factor α
TT	tetanus toxoid
VSMC	vascular smooth cell
